# An adaptive multi-modal hybrid model for classifying thyroid nodules by combining ultrasound and infrared thermal images

**DOI:** 10.1186/s12859-023-05446-2

**Published:** 2023-08-19

**Authors:** Na Zhang, Juan Liu, Yu Jin, Wensi Duan, Ziling Wu, Zhaohui Cai, Meng Wu

**Affiliations:** 1 Institute of Artificial Intelligence, School of Computer Science, Wuhan University, Wuhan, 430072 China; 2 Department of Ultrasound, Zhongnan Hospital, Wuhan University, Wuhan, 430072 China

**Keywords:** Multi-modal learning, Adaptive weight, Thyroid nodule, Infrared thermal image, Ultrasound image

## Abstract

**Background:**

Two types of non-invasive, radiation-free, and inexpensive imaging technologies that are widely employed in medical applications are ultrasound (US) and infrared thermography (IRT). The ultrasound image obtained by ultrasound imaging primarily expresses the size, shape, contour boundary, echo, and other morphological information of the lesion, while the infrared thermal image obtained by infrared thermography imaging primarily describes its thermodynamic function information. Although distinguishing between benign and malignant thyroid nodules requires both morphological and functional information, present deep learning models are only based on US images, making it possible that some malignant nodules with insignificant morphological changes but significant functional changes will go undetected.

**Results:**

Given the US and IRT images present thyroid nodules through distinct modalities, we proposed an Adaptive multi-modal Hybrid (AmmH) classification model that can leverage the amalgamation of these two image types to achieve superior classification performance. The AmmH approach involves the construction of a hybrid single-modal encoder module for each modal data, which facilitates the extraction of both local and global features by integrating a CNN module and a Transformer module. The extracted features from the two modalities are then weighted adaptively using an adaptive modality-weight generation network and fused using an adaptive cross-modal encoder module. The fused features are subsequently utilized for the classification of thyroid nodules through the use of MLP. On the collected dataset, our AmmH model respectively achieved 97.17% and 97.38% of F1 and F2 scores, which significantly outperformed the single-modal models. The results of four ablation experiments further show the superiority of our proposed method.

**Conclusions:**

The proposed multi-modal model extracts features from various modal images, thereby enhancing the comprehensiveness of thyroid nodules descriptions. The adaptive modality-weight generation network enables adaptive attention to different modalities, facilitating the fusion of features using adaptive weights through the adaptive cross-modal encoder. Consequently, the model has demonstrated promising classification performance, indicating its potential as a non-invasive, radiation-free, and cost-effective screening tool for distinguishing between benign and malignant thyroid nodules. The source code is available at https://github.com/wuliZN2020/AmmH.

## Background

Thyroid nodules are common thyroid diseases. According to epidemiological data, the incidence of thyroid nodules in the population is 19–68%, of which about 5–15% are malignant [1]. Patients diagnosed with benign nodules typically require only periodic monitoring, whereas those with malignant nodules often necessitate additional interventions. Therefore, the precise differentiation between benign and malignant nodules is crucial for effective clinical treatment planning.

In clinical practice, US is generally used for the preliminary grading of thyroid nodules because of its non-invasive, non-radiation, and low-cost characteristics [2]. Patients with TI-RADS [3] grading 4 or higher ultrasonic examination results are generally considered to be at risk of malignancy, and it is recommended that they undergo further invasive fine-needle biopsy for diagnostic purposes. Traditionally, the precise grading of thyroid nodules has relied heavily on the expertise of sonographers, who must dynamically observe various morphological and functional characteristics, including size, shape, internal structure, blood flow distribution, and hemodynamics. This approach is both inefficient and subjective. In contrast, machine learning based methods are efficient and objective, and have been widely used in fields such as disease diagnosis [4] and medical image analysis [5]. Compared with traditional machine learning methods, deep learning has the advantage of automatic learning feature representation, thus has been widely used in the medical field such as [6]. In fact, some deep learning based methods have been proposed to assist in the diagnosis of thyroid nodules by automatically classifying their US images. For example, Soon et al. [7] employed a transfer learning method with the pre-trained VGG16 model for classifying thyroid US images. Qing et al. [8] employed the Inception-v3 method to distinguish benign and malignant thyroid nodules based on US images. However, the static US images can only illustrate the size, shape, contour boundary, echo, and other morphological information of the nodules, incapable of describing the functional information such as hemodynamics and thermodynamics. In recent years, the application of the IRT technique, which has the same non-invasive, non-radiative, and low-cost advantage as US, for the detection of thyroid diseases has also been investigated by researchers. Ahdy et al. [9] detected and displayed the relative skin temperature variations of patients suffering from thyroid disorders using the IRT technique. Their analysis results showed that the IRT technique can be used to characterize thyroid nodular disease by quantifying the spatial and temporal abnormalities in skin blood perfusion. Farshad et al. [10] studied the thyroid IRT and confirmed the higher temperatures of thyroid tumors in comparison to the thyroid gland, which appears as hot spots and disturbs the symmetry of the thermogram. Based on this, they succeeded in detecting the edges of the malignant thyroid tumors in the IRT images. Viviane et al. [11] also analyzed the thermal behavior of thyroid nodules through IRT, showing the feasibility of classifying thyroid nodules using IRT images. However, the IRT images can only describe the functional information of thyroid nodules, failing to describe their morphological information. While both morphological and functional information are crucial for distinguishing between benign and malignant thyroid nodules, neither US nor IRT images alone provide a comprehensive representation of all aspects of the nodules. Currently, the development of equipment capable of capturing both morphological and functional information through US and IRT imaging remains challenging due to limitations in imaging sensors. Given the semantic correlation and complementary information provided by two distinct sources of images, it is necessary to employ the emerging multi-modal learning technique to achieve a more precise classification of thyroid nodules. However, as far as we know, there have been few such studies published.

The fusion of multi-modal data has been the research focus in the field of multi-modal learning, through which the model can benefit from different data modalities to learn complementary and supplementary information. In recent years, multi-modal learning by fusing different kinds of medical images has been used to facilitate clinical diagnosis and surgical navigation [12]. For instance, Ravi et al. [13] and Bhuyan et al. [6] proposed deep learning-based approaches for COVID-19 classification using both CT scan and chest X-ray images. Razzaghi et al. [14] proposed a multi-modal deep transfer learning for MRI brain image analysis. Li et al. [15] proposed a multi-modal fusion model based on a dense convolutional network with dual attention for PET and MRI images. Wang et al. [16] proposed a multi-modal fusion and calibration network for 3D pancreas tumor segmentation via PET and CT images. Drawing inspiration from these studies, we posit that the integration of IRT and US images through multi-modal learning can yield not only functional information from the IRT but also morphological information from the US. Consequently, we propose to combine both the US and IRT images to build the classification model of thyroid nodules based on the multi-modal learning in this paper.

Generally, there are three kinds of fusion strategies in multi-modal learning: input-level fusion, feature-level fusion, and decision-level fusion [17]. We use the feature-level fusion approach in this study to acquire complementary information from different image modalities. That is, we first extract comprehensive features from each modal data (intra-modal feature extraction) and then integrate them (inter-modal feature fusion) to build the classification model. Consequently, the initial step is to extract features to represent the original data of each modality. Convolutional neural networks (CNNs) have been extensively employed for feature extraction from input data, including medical images, since their birth, and have demonstrated remarkable proficiency in feature extraction. Nevertheless, the inability to learn long-range dependencies among features restricts CNNs from extracting only local features, which may not adequately represent the original data. To address this issue, many researchers have proposed combining other kinds of network structures to compensate for the shortcomings of CNNs. For example, Yan et al. [18] proposed a CNN-RNN (Recurrent Neural Network) hybrid network for breast cancer histopathological image classification. Ketu et al. [19] proposed a CNN-LSTM (Long Short-Term Memory) hybrid network for the prediction of the COVID-19 epidemic across India. Compared to RNNs and LSTM, the recently proposed Transformer networks are more powerful to extract information on long-range dependencies thanks to the use of the self-attention mechanism [20–23]. Moreover, Transformers have the advantages of parallelism and scalability and are not prone to gradient vanishing, making them perform excellently in many tasks. Therefore, in this paper, we present to combine a CNN and a Transformer to build a hybrid encoder (i.e., intra-modal feature extractor) for each imaging modality, to make full use of the advantages of CNNs and Transformers. In each hybrid encoder, a CNN is bridged to a Transformer through a feature embedding layer, allowing the encoder to comprehensively extract features from the corresponding modality of images.

In order to integrate features extracted from different modalities, there have several fusion strategies have been proposed, such as direct concatenating [24], fusing via the Kronecker product [25], and fusing based on orthogonalization loss [26]. However, most existing methods ignore the semantic correlation between different modalities, which may not effectively integrate information between different modalities and may introduce noise. In order to fuse complementary features of multi-modal images while retaining the unique features of different modal images, some recent researches introduce the modality-level cross-connection so that the semantic correlation between modalities can also be involved in the process of fusion [16, 27]. Nevertheless, they weigh different modalities equally in all cases (patients), which is inconsistent with the fact that different modalities may weigh differently in different cases. For example, the US images may contain more helpful information than the IRT images for some patients, while the opposite for others. Therefore, in this paper, we not only build an adaptive cross-modal encoder based on Transformer to effectively integrate correlation between different modalities but also design an adaptive modality-weight generation network to learn different weighting schemes for different cases.

To sum up, we present a novel Adaptive multi-modal Hybrid (AmmH) model to combine the US and IRT images for the classification of thyroid nodules. AmmH is designed according to the feature-level fusion strategy, therefore it mainly contains two feature extraction modules, a feature fusion module, and a classification module. The feature extraction modules are responsible for extracting features from US and IRT images respectively. The feature fusion module is adaptively integrate the features of US and IRT images to generate the comprehensive representation of a case which is then classified by the classification module. The main contributions of our work are as follows:

1. As far as we know, this work is the first attempt to combine US and IRT images for the classification of thyroid nodules. IRT and US images respectively characterize the functional and the morphological information which complements each other, so integrating these two kinds of images is expected to improve the classification performance of thyroid nodules.
2. We design a hybrid intra-modal encoder network in which the CNN is bridged to the Transformer via a feature embedding layer, so that the encoder has the advantages of both CNN and Transformer encoders, having more powerful feature extracting capability than pure CNN or Transformer encoder.
3. We design an adaptive feature fusion module consisting of a cross-modal encoder network and an adaptive weight generation network. The cross-modal encoder network can facilitate the integration of correlation between different modalities, reducing the impact of redundant and noisy features on the classification of thyroid nodules. Furthermore, the adaptive weight generation network can adaptively adjust the weights of two modal images for different cases so that our AmmH model can adaptively pay attention to different image modalities in the classification of thyroid nodules.

The remainder of this paper is organized as follows. First, we describe the details of the proposed AmmH model in the “Method” section. Then we introduce the data collection and the experiments in the “Materials and Experiments” section. Finally, we conclude our work in the “Conclusion” section.

## Method

As mentioned above, using multi-modal learning methods to jointly exploit information from multiple modalities to classify thyroid nodules has rarely been investigated. Given the significance of both morphological information represented by US images and functional information described by IRT images in distinguishing thyroid nodules, we proposed an adaptive multi-modal hybrid model, AmmH, to integrate the US and IRT images for classifying the thyroid nodules. AmmH adopts the feature-level fusion strategy to implement the multi-modal learning task. In this section, we first introduced the overview of the AmmH model. Then we described the design of its feature extraction module and the feature fusion module.

### Overview of the AmmH model

**Fig. 1:**
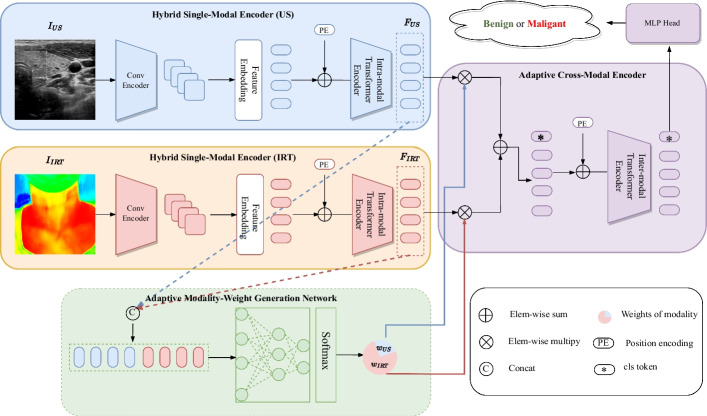
Overview of the Proposed AmmH Model. AmmH is composed of two hybrid single-modal encoders, an adaptive cross-modal encoder, and a MLP Head. Two single-modal encoders are used to extract high-level features from US images and IRT images respectively, an adaptive cross-modal encoder is used for feature fusion and the MLP Head uses the resulting features for the final thyroid nodules classification. Besides, a modality-weight generation network generates different modal weights $(\omega _{US},\omega _{IRT})$ for each case based on the features from the single-modal encoders

Figure 1 depicts the architecture of the AmmH model that consists of three main modules: the feature extraction module, the feature fusion module, and the classification module. The feature extraction module is designed as a two-branch network. Each branch is a Hybrid Single-Modal Encoder (HSME) for extracting features from the US or IRT images respectively. The feature fusion module consists of an Adaptive Modality-Weight Generation (AMWG) network and an Adaptive Cross-Modal Encoder (ACME). Based on the fact that the importance of the morphological and functional features for thyroid nodules classification may be different in different cases, the AMWG network is designed for adaptively generating the weights of two modalities, and the ACME is designed as the Transformer encoder to adaptively fuse the features of different modalities. In AmmH, we paid little attention to the design of the classification module and simply used the MultiLayer Perceptron (MLP) Head to classify the benign and malignant thyroid nodules.

As illustrated in Fig. 1, the AmmH model simultaneously accepts the US image $I_{US}$ and IRT image $I_{IRT}$ of the same patient case. The corresponding branch of HSME respectively extracts features from $I_{US}$ and $I_{IRT}$, then yields the high-level semantic features $F_{US}$ and $F_{IRT}$. which are fused in the ACME network. $F_{US}$ and $F_{IRT}$ are also fed into the AMWG network to generate the weights of two modalities, denoted as $\omega _{US}$ and $\omega _{IRT}$, so that ACME adaptively weighs different modalities in different cases. The fused features are sent to the MLP Head for the classification of thyroid nodules. The model uses a standard cross-entropy loss function to achieve end-to-end optimization in this work.

### Feature extraction module

The feature extraction module is designed as a two-branch network. Each branch is an HSME block with the same structure and is responsible for extracting both the local and global features of the corresponding modal images. The HSME is the combination of CNN and Transformer encoders and consists of three components: the intra-modal Convolutional encoder to extract the local features, the intra-modal Transformer encoder to extract the global features, and the feature embedding layer to bridge two encoders.

#### Intra-modal convolutional encoder

The intra-modal Convolutional encoder is used to generate feature maps for the US or IRT images. Though there are many CNNs that can be used as the Convolutional encoder, we chose the ResNet18 for the purpose in the current work due to the powerful feature extraction capability of the ResNet [28]. Concretely, we built the intra-modal Convolutional encoder by removing the final global pooling and full connection layers in the ResNet18. The feature maps produced by the intra-modal Convolutional encoder can be formulated as:

1 $$\begin{aligned} {F}_{m}^{\text {local }}={E}_{m}^{\text {Conv }}\left( I_{m}\right) \end{aligned}$$

where $$m\in \left\{ US,IRT \right\}$$. $$I_{m} \in R^{C * H * W}$$, $$F_{m}^{\text {local }}\in R^{C^{\prime } * H^{\prime } * W^{\prime }}$$.

#### Feature embedding layer

In order to bridge the intra-modal Convolutional encoder and the intra-modal Transformer encoder, we designed the feature embedding layer to process the feature maps obtained from the intra-modal Convolutional encoder. Using the obtained feature maps, we first performed a deconvolution operation to determine the size and number of feature maps needed. Due to Transformer’s sequence-to-sequence processing, the 2D feature maps are flattened into 1D ones. However, the ?attening operation loses spatial information, which is critical to image classification. To address this issue, we introduced the learnable position embeddings $PE_{US}$ and $PE_{IRT}$ to supplement the flattened features via element-wise summation, which is formulated as:

2 $$\begin{aligned} F_{m}^{\text {token }}=\text {Flatten}\left( {\text {Deconv}}_{m}\left( F_{m}^{\text {local }}\right) \right) +P E_{m} \end{aligned}$$

where $$m\in \left\{ US,IRT \right\}$$. $$F_{m}^{\text {token }}$$, $$P E_{m} \in R^{C^{\prime \prime } * H^{\prime \prime }W^{\prime \prime }}$$.

#### Intra-modal transformer encoder

The Convolutional encoder fails to build the long-range dependency within each modality because of some induction bias such as the translational invariance. To better capture the global information between feature maps, we designed the intra-modal Transformer encoder based on the ViT[21] to establish the long-range dependencies. The encoder consists of four intra-modal Transformer blocks, each block contains a Self-Attention (*SA*) [20], and a MLP. In the *SA*, each token does an attentional calculation with all surrounding tokens, so that the long-range dependencies can be captured. The *SA* can be formulated as:

3 $$\begin{aligned} Q_{m}= & {} W_{m}^{Q} * L N\left( F_{m}^{\text {token }}\right) \end{aligned}$$

4 $$\begin{aligned} K_{m}= & {} W_{m}^{K} * L N\left( F_{m}^{\text {token }}\right) \end{aligned}$$

5 $$\begin{aligned} V_{m}= & {} W_{m}^{V} * L N\left( F_{m}^{\text {token }}\right) \end{aligned}$$

6 $$\begin{aligned} S A_{m}= & {} {\text {softmax}}\left( \frac{Q_{m} K_{m}^{T}}{\sqrt{d_{k}}}\right) V_{m} \end{aligned}$$

where $m\in \left\{ US,IRT \right\}$. $W_{m}^{Q}$, $W_{m}^{K}$ and $W_{m}^{V}$ are learnable parameters. $L N\left( \cdot \right)$ is Layer Normalization [29]. $d_{k}$ is the dimension of $K_{m}$. The MLP is a 2-layer Linear with GELU [30].

Therefore, the features with the local context information and global context information within each modality produced by the HSME can be defined as:

7 $$\begin{aligned} {x}_{m}= & {} {S} A_{m}\left( {L N}\left( {F}_{m}^{\text {token }}\right) \right) +{F}_{m}^{\text {token }} \end{aligned}$$

8 $$\begin{aligned} {F}_{m}= & {} {M L P _ { m }}\left( {L N}\left( x_{m}\right) \right) +x_{m} \end{aligned}$$

where $$m\in \left\{ US,IRT \right\}$$. $$F_{m} \in R^{C^{\prime \prime } * H^{\prime \prime }W^{\prime \prime }}$$.

### Feature fusion module

The feature fusion module aims to adaptively establish long-range correlation across modalities for modality-invariant features with global semantics. It efficiently integrates information from different modalities and learns adaptively for different cases, giving different weighing schemes for different cases. This module is composed of an adaptive modality-weight generation network and an adaptive cross-modal encoder network.

#### Adaptive modality-weight generation network

In an ideal multi-modal model, though all available information from different modalities would be used to make the prediction, the model should be able to adaptively emphasize one modality over another according to the specific patient case. To achieve this, we designed the AMWG network to adaptively generate the optimal weights of different modalities for accurate classification. AMWG accepts the high-level semantic features generated by the HSME network instead of the raw images in order to reduce the repetitive computation and accelerate convergence. Moreover, using the features as the inputs also forces the AMWG to allocate weights explicitly based on discriminative features and to coordinate parameters updates across modules. In this work, the AMWG is designed as an MLP containing three linear layers with the ReLU activation. It should be noticed that the AMWG network is part of the multi-modal learning framework (Fig. 1), therefore the weight parameters of AMWG, together with those of other parts of the multi-modal learning framework, are dynamically updated by minimizing the loss during the model training process. Based on the weight parameters in AMWG, we can generate the modality weight for each modality using the following equation:

9 $$\begin{aligned} \left( \omega _{U S}, \omega _{IRT}\right) ={\text {softmax}}\left( M L P_{A M W G}\left( \left[ F_{U S}, F_{I R T}\right] \right) \right) \end{aligned}$$

where $\left[ \cdot ,\cdot \right]$ is concatenation operation. The purpose of using the *softmax* function here is to ensure that the sum of different modality feature weights must be 1, *i.e.*, $\omega _{U S}+\omega _{I R T}=1$.

#### Adaptive cross-modal encoder

We implemented the ACME also based on the Transformer blocks in ViT [21]. To distinguish from the intra-modal Transformer in HSME, it is called as inter-modal Transformer here. The inter-modal Transformer encoder combines the features generated from two HSME encoders by weighted summation as the multi-modal token. Moreover, a *cls* token [31] is introduced for the final classification of thyroid nodules. Therefore, the final input $F^{\text {token }}$ of the inter-modal Transformer encoder is defined as:

10 $$\begin{aligned} F^{\text {token }}=\left[ cls \ \text {token, } w_{U S} * F_{U S}+w_{I RT} * F_{I RT}\right] +\textrm{PE} \end{aligned}$$

where ${F}^{\text {token }} \in {R}^{\left( c^{\prime \prime }+1\right) * {H}^{\prime \prime } W^{\prime \prime }}$ , *PE* is learnable position embedding.

**Fig. 2:**
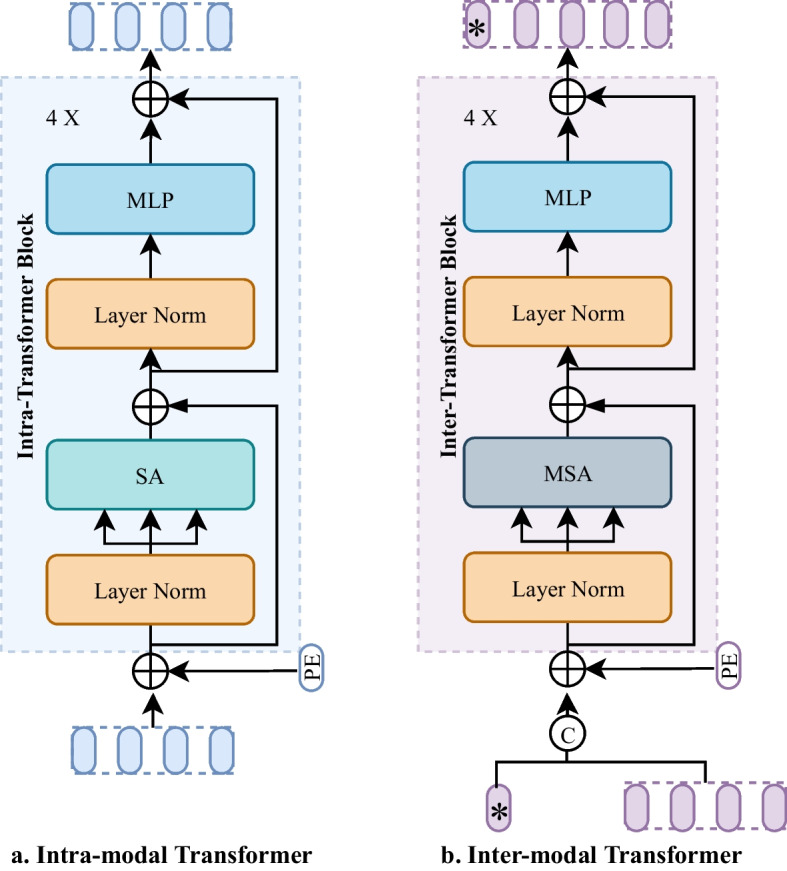
Comparison of the Intra-Modal Transformer in HSME for Feature Extraction and the Inter-Modal Transformer in ACME for Feature Fusion. **a** Illustrates the intra-modal Transformer, and **b** illustrates the inter-modal Transformer

Besides, in order to allow the model to learn information from both modalities in several different representation subspaces for better information interaction, we used a Multi-headed Self-Attention (*MSA*) mechanism [20] in the inter-modal Transformer encoder, which is different from the intra-modal Transformer block where the *SA* mechanism is used. The *MSA* is defined as:

11 $$\begin{aligned}{} & {} Q^{i}=W^{Q^{i}} \textbf{L N}\left( F^{\text {token }}\right) \end{aligned}$$

12 $$\begin{aligned}{} & {} K^{i}=W^{K^{i}} \textbf{L N}\left( F^{\text {token }}\right) \end{aligned}$$

13 $$\begin{aligned}{} & {} V^{i}=W^{V^{i}} \textbf{L N}\left( F^{\text {token }}\right) \end{aligned}$$

14 $$\begin{aligned}{} & {} \text {head }^{i}=S A^{i}={\text {softmax}}\left( \frac{Q^{i} * K^{i}}{\sqrt{d_{k}}}\right) V^{i} \end{aligned}$$

15 $$\begin{aligned}{} & {} \text {MSA }=W^{\textrm{o}}\left[ \text {head }^{1}, \text {head }^{2} \ldots \text {head }^{N}\right] \end{aligned}$$

16 $$\begin{aligned}{} & {} x=M S A\left( L N\left( F^{\text {token }}\right) \right) +F^{\text {token }} \end{aligned}$$

17 $$\begin{aligned}{} & {} F={\text {MLP}}(L N(x))+x \end{aligned}$$

where the head number is 8 in our implementation. The final output *cls* token is sent to the MLP Head for the final thyroid nodules classification. Figure 2 shows the difference between the intra-modal Transformer and the inter-modal Transformer. Figure 2a illustrates the intra-modal Transformer, and Fig. 2b illustrates the inter-modal Transformer. The main difference between them is the attention mechanism. The self-attention is used in the intra-modal Transformer and multi-headed attention is used in the inter-modal Transformer. In addition, a *cls* token(*) is introduced in the inter-modal Transformer for the final classification of thyroid nodules.

## Materials and experiments

### Data collection

To the best of our knowledge, there are no similar studies that combine US images with IRT images to assist in the diagnosis of thyroid nodules. Since we can not find any public dataset that contains both US and IRT images of the thyroid nodules, we constructed a dataset, Th-USIRT, to validate our proposed method.

**Fig. 3:**
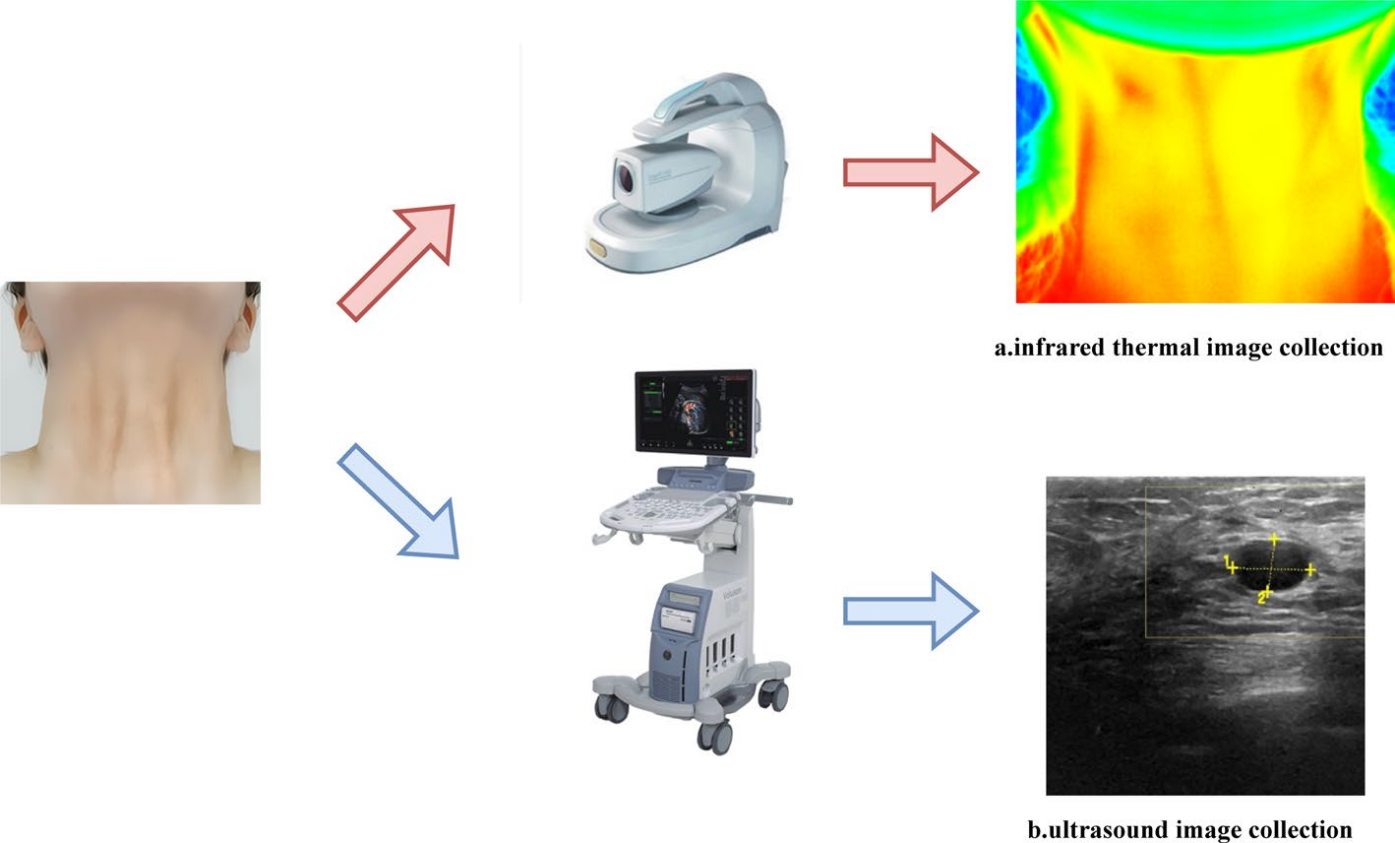
Flow Chart of the Image Acquisition. **a** Illustrates the flow of IRT image collection and **b** Illustrates the flow of US image collection

The study subjects were patients who underwent thyroid ultrasonography at the partner hospital from October 2021 to September 2022. Besides the US images, we also acquired the IRT images of their neck areas using the HB-T-1 Thermal Imaging System. The device uses an uncooled infrared focal detector with $320 \times 240$ pixel chip, temperature resolution of $0.08\,^{\circ }\textrm{C}$, and temperature range of 20–$40\,^{\circ }\textrm{C}$. The subject entered the examination room, removed the wearing apparel, exposed the neck, and sat quietly for 3–5 min on the chair to sufficiently dissipate the heat, keep quiet, and stable the temperature of the area to be examined. The examiner acquired the IRT image by placing the device’s probe squarely on the subject’s neck so that the neck is in the middle of the image, with the upper screenshot of the image at ear level (containing the entire neck) and the lower screenshot of the image at shoulder level. As soon as the IRT images had been acquired, the subjects would undergo thyroid ultrasonography and the US images can also be collected. Figure 3 illustrates the flow chart of the image acquisition.

**Fig. 4:**
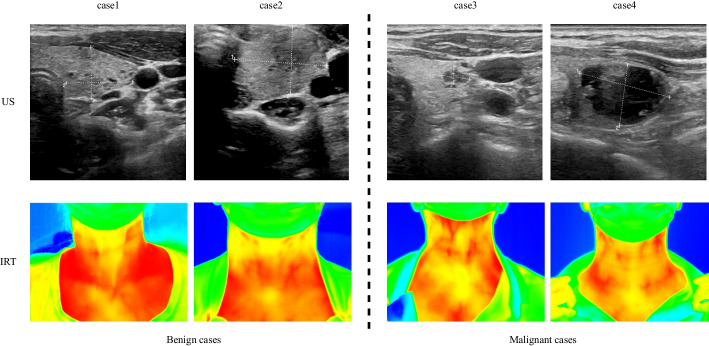
Example Images in the Th-USIRT Dataset. The left are cases of benign nodules and the right are cases of malignant nodules

In this study, we have collected the thyroid US and IRT images of 2864 patients having the diagnostic results. The resolution of each US image is $512\times 512$ and the resolution of each IRT image is $320 \times 240$. According to the diagnostic results, the thyroid nodules with TI-RADS [3] grading 4 and above are considered as malignant and others are considered as benign. As a result, 1536 pairs of US and IRT images are labeled as benign, and 1328 pairs are labeled as malignant. All image pairs and their labels were collected to form the dataset Th-USIRT. Figure 4 exemplifies the US and IRT images in the dataset. The dataset has been randomly divided into the training, validation, and test sets by the ratio of 6:2:2. Table 1 presents the number of image pairs in different sets.

**Table 1:** The number of image pairs in different sets

	Training set	Validation set	Test set	Total number
Benign	922	307	307	1536
Malignant	798	265	265	1328

### Experimental settings

To evaluate our method, we performed two kinds of experiments based on the Th-USIRT dataset. It should be noted that all images of both modalities are uniformly reshaped to $224\times 224$ in all experiments. First, we compared the AmmH with several deep learning methods that have been applied to assist in the diagnosis of thyroid nodules. Now that the compared methods are all single-modal, we trained and tested two models for each of these methods separately using the US images and the IRT images. While we trained and tested our method using two kinds of images. Then, we conducted four kinds of ablation studies to confirm the advantages of different modules in our proposed AmmH model. Finally, we conducted an analysis of the complexity of our models.

All experiments were conducted on a computer configured with 8 GeForce RT, and X 3090Ti GPUs and all models were implemented in PyTorch. As mentioned above, we selected ResNet [28] as the CNN block and ViT [21] as the Transformer blocks in the current implementation of our AmmH model, since they are the most popular models for image classification tasks. In this work, we did not pay much attention to the design of the loss function and simply adopted the commonly used cross-entropy loss function though other loss functions can also be considered. During the training process, the Adam optimizer was utilized to minimize the categorical cross-entropy loss. Other parameter settings are revealed in Table 2. The Accuracy(ACC), Sensitivity (SEN), Precision (PRE), Specificity (SPE), F1-score, and F2-score [32] were utilized to evaluate the performance of the models.

**Table 2:** Configuration of the training parameters of the model

Param	Value
Batch size	32
Epoch	200
Initial learning rate/min learning rate	1e−3 / 1e−4
Learning decay type	cos
$$\beta$$	(0.9, 0.999)
weight_decay	5e−4

### Comparison AmmH with other methods

In this experiment, we compared our AmmH with four single-modal deep learning approaches that had successfully been applied to assist in the diagnosis of thyroid nodules: VGG16, Inception V3, ResNet18, and ViT. Each of the single-modal methods was trained respectively using the US and IRT images to generate two classification models. Whereas our multi-modal AmmH was trained with US and IRT images at the same time to generate one classification model. The comparison results of nine models are listed in Table 3. In this table, we can see that the classification performance of our AmmH model is significantly better than the others.

We think the reasons why AmmH is superior to others can be attributed to the following three aspects. The first one is the use of multi-modal data. Data from different modalities can provide information from different perspectives, which complement each other so that the fused information is more comprehensive to represent the thyroid nodules. The second one is the design of the HSME block in the feature extraction module. HSME combines the advantages of CNNs for local feature extraction and Transformer for global feature modeling, which enables our model to extract more powerful features from the data. The third one is the design of the feature fusion module containing ACME and AMWG. ACME uses the Transformer to fuse features from different modalities to enable the learning of correlations of different modalities, reducing the noise and redundancy. Moreover, the AMWG network allows our model to pay adaptive attention to different modalities according to different cases so that the personalized feature representations that are most conducive to the classification of their thyroid nodules can be learned.

**Table 3:** Comparison of methods for classification of thyroid nodules

Row	Modality	Model	ACC	PRE	SEN	SPE	F1	F2
1	US	VGG	0.6838	0.7410	0.4881	0.8527	0.5885	0.7419
2	US	ResNet	0.7570	0.8316	0.5962	0.8958	0.6945	0.8140
3	US	Inception	0.7298	0.6744	0.8056	0.6644	0.7342	0.6885
4	US	ViT	0.7395	0.7710	0.6226	0.8404	0.6889	0.7854
5	IRT	VGG	0.7188	0.7705	0.5595	0.8562	0.6483	0.7741
6	IRT	ResNet	0.7098	0.7463	0.5660	0.8339	0.6438	0.7618
7	IRT	Inception	0.7482	0.7366	0.7103	0.7808	0.7232	0.7154
8	IRT	ViT	0.7378	0.7138	0.7245	0.7492	0.7191	0.7441
9(ours)	US + IRT	AmmH	**0.9738**	**0.9699**	**0.9736**	**0.9739**	**0.9717**	**0.9738**

Bold values indicate the best results achieved in each indicator

### Ablation study on multi-modal learning

In order to investigate whether the multi-modal learning strategy to combine the US and IRT images helps to improve the classification performance, we constructed three multi-modal models based on our AmmH. Specifically, we removed the ACME module as well as the AMWG network block in AmmH. The features extracted from two branches were directly concatenated for the downstream classification task. We call this modified model as “Hybrid w/o ACME”. We further modified the HSME block in the “Hybrid w/o ACME” model by cutting off one of the intra-modal Transformer encoder and intra-modal Convolutional encoder to get two multi-modal models with pure CNN and Transformer, called “ResNet w/o AMCE” and “ViT w/o AMCE” respectively. Besides, we deleted one HSME branch and the feature fusion module in “Hybrid w/o ACME” to obtain a single-modal model, called “Hybrid”. Accordingly, we compared ResNet with “ResNet w/o AMCE”, ViT with “ViT w/o AMCE”, and “Hybrid” with ‘Hybrid w/o ACME”. The comparing results are presented in Table 4.

According to Table 4, it is obvious that the multi-modal learning models based on the same network backbone have significantly higher classification performance than the single-modal learning models, which demonstrates that the joint consideration of US and IRT images using multi-modal learning is necessary to accurately classify the thyroid nodules. The US images reflect the morphological characteristics of the thyroid nodules in the area of the lesion while the IRT images reflect the thermodynamic characteristics of the thyroid nodules. Multi-modal learning helps to extract different features from different views, contributing to the diversity of data representation and the strong discriminative abilities of the models. Furthermore, when the data of one modality is disturbed by noise, the information provided by other modalities can assist in correcting it and the integrated noise does not synchronize the consistent information in the data of different modalities so that the accuracy and robustness of decision-making can be improved.

**Table 4:** Comparison of classification performance of different models with difference encoders

Modality	Model	ACC	PRE	SEN	SPE	F1	F2
US	ResNet	0.7570	0.8316	0.5962	0.8958	0.6945	0.8140
IRT	ResNet	0.7098	0.7463	0.5660	0.8339	0.6438	0.7618
US+IRT	ResNet w/o AMCE	**0.8444**	**0.9000**	**0.7472**	**0.9283**	**0.8165**	**0.8854**
US	ViT	0.7395	0.7710	0.6226	**0.8404**	0.6889	0.7854
IRT	ViT	0.7378	0.7138	0.7245	0.7492	0.7191	0.7441
US+IRT	ViT w/o AMCE	**0.8357**	**0.8178**	**0.8302**	**0.8404**	**0.8240**	**0.8383**
US	Hybrid	0.7640	0.8009	0.6528	0.8599	0.7193	0.8086
IRT	Hybrid	0.7483	0.8040	0.6038	0.8730	0.6897	0.8015
US+IRT	Hybrid w/o AMCE	**0.8636**	**0.8880**	**0.8075**	**0.9121**	**0.8458**	**0.8891**

Bold values indicate the best results achieved in each indicator

“w/o” indicates “without”; “ACME” indicates the adaptive cross-modal encoder

### Ablation study on HSME block

**Fig. 5:**
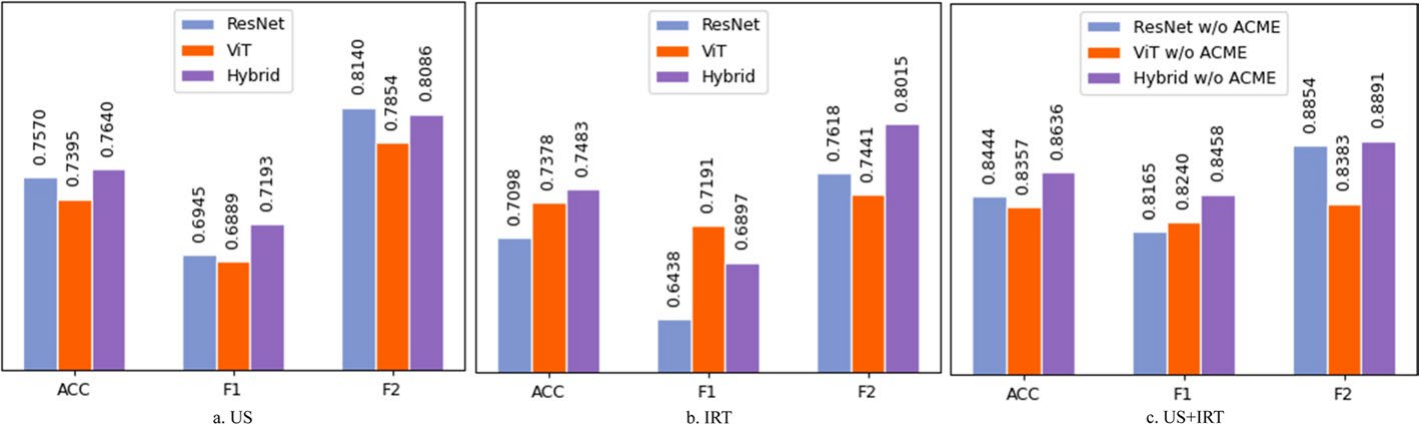
Comparison Results of Models with Hybrid and Pure Feature Encoders. **a** On US, **b** on IRT, **c** on US and IRT

To do the ablation study on the HSME block, we compared the models with pure CNN/Transformer feature extractors and with hybrid (i.e., HSME) feature extractors. As shown in Fig. 5, the models using the HSME block achieved the highest accuracies, and generally performed better than those extracting features via pure CNN or Transformer encoders.

It is interesting to find that the US single-modal models with pure CNN feature extractors performed slightly better than those with pure Transformer feature extractors, whereas the IRT single-modal models with pure Transformer feature extractors performed slightly better than those with pure CNN feature extractors. This phenomenon suggests that CNN still has a significant role to play in the extraction of features from images, although the Transformer-based models have been developing rapidly in the field of computer vision over the past 2 years. Different encoders behave differently to the characteristics of the images of different modalities. A model needs to focus on different features of different modalities using different feature extractors. For example, if it is needed to classify the thyroid nodules using IRT images, the model should be more concerned with the temperature distribution characteristics, which means that it should have a good global feature modeling capability. If the US images are used, the model should focus on local features such as the boundary and texture of the lesion area, which means that it should have good local feature extraction capability. Therefore, CNN and Transformer hybrid encoder should be used in the feature extraction phase in our multi-modal models, which has been confirmed by the results of the ablation experiments.

### Ablation study on ACME block

In order to explore the capability of the ACME component in multi-modal feature fusion, we compared three pairs of multi-modal models. The only difference between the two models in each pair is whether the ACME is used. Table 5 lists the comparing results. As we can see in Table 5, the introduction of ACME has improved the accuracy of the multi-modal approaches by over 10%. ACME processes and integrates the extracted features from different modalities based on the Transformer module, which weakens the effects of redundant features and some noise from two different modalities, and realizes sufficient information interaction between the modalities. Therefore, the introduction of ACME greatly improves the performance of the model. The experimental results also demonstrate that the combination of ACME and HSME can provide richer information for the classification of thyroid nodules, which makes our AmmH model outperform all other competing multi-modal methods.

**Table 5:** Comparison results of multi-modal models with (without) ACME

Model	ACC	PRE	SEN	SPE	F1	F2
ResNet w/o AMCE	0.8444	0.9000	0.7472	0.9283	0.8165	0.8854
ResNet w/ AMCE	**0.9406**	**0.9358**	**0.9358**	**0.9446**	**0.9358**	**0.9428**
ViT w/o AMCE	0.8357	0.8178	0.8302	0.8404	0.8240	0.8383
ViT w/ AMCE	**0.9476**	**0.9242**	**0.9660**	**0.9316**	**0.9446**	**0.9383**
Hybrid w/o AMCE	0.8636	0.8880	0.8075	0.9121	0.8458	0.8891
AmmH	**0.9738**	**0.9699**	**0.9736**	**0.9739**	**0.9717**	**0.9738**

Bold values indicate the best results achieved in each indicator

“w/” indicates “with”; “w/o” indicates “without”; “ACME” indicates adaptive cross-modal encoder

### Ablation study on AWMG block

To investigate whether the AWMG is helpful to improve the performance of our model, we removed this block from AmmH and compared it with the original AmmH. It can be observed in Table 6 that the introduction of AWMG can actually boost the model’s performance by enabling personalized weighting for different modalities. This is due to the fact that there are significant differences between individual cases, and the model should adaptively assign different weights to each modality for different cases when making the final decision, rather than treating all modalities equally. Note that as the design of AWMG is general, it could be easily extended to other multi-modal problems in future applications.

**Table 6:** Comparison of experimental results with (without) adaptive modality-weight generation component in AmmH model

Row	model	ACC	PRE	SEN	SPE	F1	F2
1(ours)	AmmH	**0.9738**	**0.9699**	0.9736	**0.9739**	**0.9717**	**0.9738**
2	AmmH w/o AWMG	0.9545	0.9164	**0.9925**	0.9218	0.9529	0.9351

Bold values indicate the best results achieved in each indicator

“w/o” indicates “without”; “AWMG” indicates adaptive weight-modality generation

### Complexity analysis

In order to better evaluate the various models, we analyzed their complexity, and the results are shown in Table 7. It reveals that multi-modal models, in comparison to their single-modal counterparts, exhibit enhanced classification performance at the expense of increased time and space complexity, which is attributed to the need for an additional feature extractor for each supplementary modality. We can also see that the pure Transformer-based multi-modal models (ViT) do not significantly perform better than the pure CNN-based multi-modal models (ResNet), though the time and space complexity of the former is considerably higher than the latter, suggesting that the elevated complexity does not necessarily ensure the superior classification performance. It is noticeable that the time and space complexity of our proposed AmmH model (a hybrid of CNN and Transformer) is just about half of the pure-Transformer based model, while a little higher than that of the pure CNN-based model. Nevertheless, AmmH performs significantly better than the above two models. This further confirms that although both CNN and Transformer have shortcomings, they have complementarity. Therefore, the hybrid of them can simultaneously have the local feature learning ability of CNN and the global feature learning ability of Transformer, thus achieving excellent classification performance in our work. Considering the fact that the complexity of CNN is much lower than that of Transformer, we believe that our AmmH model can well balance the complexity and classification performance.

**Table 7:** Classification performance versus model complexity

Modality	Model	F1	F2	Params^a^	FLOPs^b^
Single(US)	ResNet	0.6945	0.8140	11.17 M^c^	1.96 G^d^
Single(IRT)	ResNet	0.6438	0.7618	11.17 M	1.96 G
Single(US)	ViT	0.6889	0.7854	85.65 M	16.86 G
Single(IRT)	ViT	0.7191	0.7441	85.65 M	16.86 G
Two(US+IRT)	ResNet w/ AMCE	0.9358	0.9428	87.58 M	16.47 G
Two(US+IRT)	ViT w/ AMCE	0.9446	0.9383	236.54 M	46.28 G
Two(US+IRT)	AmmH(Hybrid w/ AMCE)	0.9717	0.9738	121.30 M	23.77 G

^a^ The number of parameters that need to be trained during the model training, which is used to measure the space complexity of a model.

^b^ The number of floating-point operations, which is used to measure the time complexity of a model.

^c^ M: Millions.

^d^ G: 10$$^9$$

## Conclusion

In this paper, we propose a novel AmmH model for classifying thyroid nodules using US and IRT images. The AmmH consists of a two-branch feature extraction module, each branch of which is a Hybrid Single-modal Encoder (HSME) by bridging CNN and Transformer to extract the local and global features of a single modality, and an adaptive feature module that can encourage interactions and build long-range dependencies between different modalities via an ACME component with adaptive weights for different cases through the AWMG network. The design of our model is general and could be applied to other multi-modal applications. We validated our method on our Th-USIRT dataset. The experimental results showed that the multi-modal methods outperformed the single-modal methods for classifying the thyroid nodules, and the CNN-Transformer hybrid feature extractors had better feature extraction abilities than pure CNN or pure Transformer encoders. Besides, the introduction of the ACME allows the model to better fuse information from US and IRT images, providing richer information helpful for the classification of thyroid nodules. Our model, AmmH, outperformed all other competing methods, suggesting that it is a suitable method for our task. However, there is still room for improvement in the balance between performance and complexity of our model. For instance, we adopted the same encoders (i.e., HSME) to respectively extract features from US and IRT images without designing more suitable feature extractors for different image modalities. In the future, we will further study the characteristics of different image modalities and design feature extractors with lower complexity while ensuring the classification performance of the multi-modal models. Despite some limitations, overall, the proposed approach still holds significant potential in the automated and accurate diagnosis of thyroid nodules.

## Data Availability

The data that support the findings of this study are available on request from the author [NZ]. The data are not publicly available due to them containing information that could compromise research participant consent. The code is open-source and can be found in the GitHub project repository:https://github.com/wuliZN2020/AmmH.

## References

[1] Abbasian Ardakani A, Bitarafan-Rajabi A, Mohammadzadeh A, Mohammadi A, Riazi R, Abolghasemi J, Homayoun Jafari A, Bagher Shiran M (2019). A hybrid multilayer filtering approach for thyroid nodule segmentation on ultrasound images. J Ultrasound Med.

[2] Burman KD, Wartofsky L (2015). Thyroid nodules. N Engl J Med.

[3] Russ G (2016). Risk stratification of thyroid nodules on ultrasonography with the french ti-rads: description and reflections. Ultrasonography.

[4] Dash TK, Chakraborty C, Mahapatra S, Panda G (2022). Gradient boosting machine and efficient combination of features for speech-based detection of covid-19. IEEE J Biomed Health Inform.

[5] Tournier J-D, Smith R, Raffelt D, Tabbara R, Dhollander T, Pietsch M, Christiaens D, Jeurissen B, Yeh C-H, Connelly A (2019). Mrtrix3: a fast, flexible and open software framework for medical image processing and visualisation. Neuroimage.

[6] Bhuyan HK, Chakraborty C, Shelke Y, Pani SK (2022). Covid-19 diagnosis system by deep learning approaches. Expert Syst.

[7] Kwon SW, Choi IJ, Kang JY, Jang WI, Lee G-H, Lee M-C (2020). Ultrasonographic thyroid nodule classification using a deep convolutional neural network with surgical pathology. J Digit Imaging.

[8] Guan Q, Wang Y, Du J, Qin Y, Lu H, Xiang J, Wang F (2019). Deep learning based classification of ultrasound images for thyroid nodules: a large scale of pilot study. Ann Transl Med.

[9] Helmy A, Holdmann M, Rizkalla M (2008). Application of thermography for non-invasive diagnosis of thyroid gland disease. IEEE Trans Biomed Eng.

[10] Bahramian F, Mojra A (2020). Thyroid cancer estimation using infrared thermography data. Infrared Phys Technol.

[11] de Camargo VMB, Ulbricht L, Coninck JCP, Ripka WL, Gamba HR (2022). Thermography as an aid for the complementary diagnosis of nodules in the thyroid gland. Biomed Eng Online.

[12] Tang W, He F, Liu Y, Duan Y (2022). Matr: multimodal medical image fusion via multiscale adaptive transformer. IEEE Trans Image Process.

[13] Ravi V, Narasimhan H, Chakraborty C, Pham TD (2022). Deep learning-based meta-classifier approach for covid-19 classification using ct scan and chest x-ray images. Multimedia Syst.

[14] Razzaghi P, Abbasi K, Shirazi M, Rashidi S (2022). Multimodal brain tumor detection using multimodal deep transfer learning. Appl Soft Comput.

[15] Li B, Hwang J-N, Liu Z, Li C, Wang Z (2022). Pet and mri image fusion based on a dense convolutional network with dual attention. Comput Biol Med.

[16] Wang F, Cheng C, Cao W, Wu Z, Wang H, Wei W, Yan Z, Liu Z (2023). Mfcnet: a multi-modal fusion and calibration networks for 3d pancreas tumor segmentation on pet-ct images. Comput Biol Med.

[17] Huang W, Wang X, Huang Y, Lin F, Tang X (2022) Multi-parametric magnetic resonance imaging fusion for automatic classification of prostate cancer. In: 2022 44th Annual international conference of the ieee engineering in medicine & biology Society (EMBC), pp. 471–474. IEEE. 10.1109/EMBC48229.2022.9871334 36085623

[18] Yan R, Ren F, Wang Z, Wang L, Zhang T, Liu Y, Rao X, Zheng C, Zhang F (2020). Breast cancer histopathological image classification using a hybrid deep neural network. Methods.

[19] Ketu S, Mishra PK (2022). India perspective: Cnn-lstm hybrid deep learning model-based covid-19 prediction and current status of medical resource availability. Soft Comput.

[20] Vaswani A, Shazeer N, Parmar N, Uszkoreit J, Jones L, Gomez AN, Kaiser L, Polosukhin I (2017) Attention is all you need. In: Guyon, I., Luxburg, U., Bengio, S., Wallach, H., Fergus, R., Vishwanathan, S., Garnett, R. (eds.) Advances in neural information processing systems 30 (NIPS 2017). Advances in Neural Information Processing Systems, vol. 30 (2017). In: 31st Annual Conference on Neural Information Processing Systems (NIPS), Long Beach, CA, DEC 04-09.

[21] Dosovitskiy A, Beyer L, Kolesnikov A, Weissenborn D, Zhai X, Unterthiner T, Dehghani M, Minderer M, Heigold G, Gelly S et al: (2020) An image is worth 16x16 words: transformers for image recognition at scale. In: International Conference on Learning Representations.

[22] Touvron H, Cord M, Douze M, Massa F, Sablayrolles A, Jégou H (2021) Training data-efficient image transformers & distillation through attention. In: International conference on machine learning, pp. 10347–10357 . PMLR.

[23] Liu Z, Lin Y, Cao Y, Hu H, Wei Y, Zhang Z, Lin S, Guo B (2021) Swin transformer: Hierarchical vision transformer using shifted windows. In: Proceedings of the IEEE/CVF international conference on computer vision, pp. 10012–10022.

[24] Mobadersany P, Yousefi S, Amgad M, Gutman DA, Barnholtz-Sloan JS, Velázquez Vega JE, Brat DJ, Cooper LA (2018). Predicting cancer outcomes from histology and genomics using convolutional networks. Proc Natl Acad Sci.

[25] Chen RJ, Lu MY, Wang J, Williamson DF, Rodig SJ, Lindeman NI, Mahmood F (2020). Pathomic fusion: an integrated framework for fusing histopathology and genomic features for cancer diagnosis and prognosis. IEEE Trans Med Imag.

[26] Braman N, Gordon JW, Goossens ET, Willis C, Stumpe MC, Venkataraman J (2021) Deep orthogonal fusion: Multimodal prognostic biomarker discovery integrating radiology, pathology, genomic, and clinical data. In: International conference on medical image computing and computer-assisted intervention, pp. 667–677. Springer.

[27] Zhou T (2023). Modality-level cross-connection and attentional feature fusion based deep neural network for multi-modal brain tumor segmentation. Biomed Signal Process Control.

[28] He K, Zhang X, Ren S, Sun J (2016) Deep residual learning for image recognition. In: Proceedings of the IEEE conference on computer vision and pattern recognition, pp. 770–778.

[29] Ba JL, Kiros JR, Hinton GE. Layer normalization stat. 2016;1050:21.

[30] Hendrycks D, Gimpel K (2016) Bridging nonlinearities and stochastic regularizers with gaussian error linear units.

[31] Kenton JDM-WC, Toutanova LK (2019) Bert: Pre-training of deep bidirectional transformers for language understanding. In: Proceedings of NAACL-HLT, pp. 4171–4186.

[32] Devarriya D, Gulati C, Mansharamani V, Sakalle A, Bhardwaj A (2020). Unbalanced breast cancer data classification using novel fitness functions in genetic programming. Expert Syst Appl.

